# XFEL Crystal Structures of Peroxidase Compound II

**DOI:** 10.1002/ange.202103010

**Published:** 2021-05-19

**Authors:** Hanna Kwon, Jaswir Basran, Chinar Pathak, Mahdi Hussain, Samuel L. Freeman, Alistair J. Fielding, Anna J. Bailey, Natalia Stefanou, Hazel A. Sparkes, Takehiko Tosha, Keitaro Yamashita, Kunio Hirata, Hironori Murakami, Go Ueno, Hideo Ago, Kensuke Tono, Masaki Yamamoto, Hitomi Sawai, Yoshitsugu Shiro, Hiroshi Sugimoto, Emma L. Raven, Peter C. E. Moody

**Affiliations:** ^1^ School of Chemistry University of Bristol Cantock's Close Bristol BS8 1TS UK; ^2^ Department of Molecular and Cell Biology and Leicester Institute of Structural and Chemical Biology University of Leicester Lancaster Road Leicester LE1 7RH UK; ^3^ Centre for Natural Products Discovery, Pharmacy and Biomolecular Sciences Liverpool John Moores University James Parsons Building, Byrom Street Liverpool L3 3AF UK; ^4^ RIKEN SPring-8 Center 1-1-1 Kouto Sayo Hyogo 679-5148 Japan; ^5^ Present address: MRC Laboratory of Molecular Biology Francis Crick Avenue, Cambridge Biomedical Campus Cambridge CB1 0QH UK; ^6^ Japan Synchrotron Radiation Research Institute 1-1-1 Kouto Sayo Hyogo 679-5198 Japan; ^7^ Graduate School of Life Science University of Hyogo 3-2-1 Kouto, Kamigori-cho Ako-gun Hyogo 678-1297 Japan

**Keywords:** heme, heme proteins, peroxidase

## Abstract

Oxygen activation in all heme enzymes requires the formation of high oxidation states of iron, usually referred to as ferryl heme. There are two known intermediates: Compound I and Compound II. The nature of the ferryl heme—and whether it is an Fe^IV^=O or Fe^IV^‐OH species—is important for controlling reactivity across groups of heme enzymes. The most recent evidence for Compound I indicates that the ferryl heme is an unprotonated Fe^IV^=O species. For Compound II, the nature of the ferryl heme is not unambiguously established. Here, we report 1.06 Å and 1.50 Å crystal structures for Compound II intermediates in cytochrome *c* peroxidase (C*c*P) and ascorbate peroxidase (APX), collected using the X‐ray free electron laser at SACLA. The structures reveal differences between the two peroxidases. The iron‐oxygen bond length in C*c*P (1.76 Å) is notably shorter than in APX (1.87 Å). The results indicate that the ferryl species is finely tuned across Compound I and Compound II species in closely related peroxidase enzymes. We propose that this fine‐tuning is linked to the functional need for proton delivery to the heme.

## Graphical abstract

Enzymatic fine‐tuning of heme reactivity: Free‐electron laser crystal structures of two different heme‐containing peroxidases—cytochrome *c* peroxidase and ascorbate peroxidase—show differences in the nature of the ferryl species. Precise enzymatic fine‐tuning within structurally similar heme active sites is implicated.

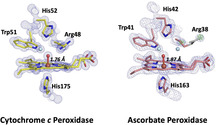

## Introduction

A large number of heme enzymes use ferryl heme during catalysis. Ferryl heme refers broadly to a highly oxidized form of the heme in which the iron is in an oxidation state that is either one equivalent (Fe^IV^, known as Compound II) or two equivalents (formally Fe^V^, known as Compound I) above the resting oxidation state (Fe^III^). The second oxidizing equivalent in Compound I resides on the porphyrin ring or, in some cases, on a protein radical.^[1]^

These Compound I and Compound II species were identified in the late 1930s^[2]^ and were later given their names.^[3]^ Because these intermediates are used in such a wide range of catalytic enzymes, they continue to attract attention these many years later.^[4]^ The chemical nature of the ferryl species has therefore been a matter of intense interest. The discussion has often focussed on whether the ferryl species is best formulated as an unprotonated Fe^IV^=O or a protonated Fe^IV^‐OH species. This matters because the protonation state controls the reactivity. The majority of the early spectroscopy on these intermediates was carried out on one of the more experimentally amenable peroxidases (usually horseradish peroxidase or cytochrome *c* peroxidase), or on myoglobin, each of which contain a histidine as proximal ligand. Most, but not all, of this information favoured an unprotonated Fe^IV^=O species for Compound I.^[5]^ But the difficulty of the experiments on such unstable species, coupled with the problems associated with generating them in quantitative yields and sometimes in high concentrations, meant that the literature was inconclusive.

From about 2000 onwards, the field moved away from spectroscopy and X‐ray crystallographic work became feasible instead. Since protons are not normally visible at the resolutions typically observed for protein crystal structures, the question instead became focussed on the iron‐oxygen bond length which was taken as an indirect reporter of bond order (single or double). Protonation state (Fe^IV^=O or Fe^IV^‐OH) is thus inferred from the bond length measured in the structure. But the picture did not clarify when X‐ray structures of ferryl species emerged, because the structures revealed bond lengths that were longer than would be expected for a pure Fe^IV^=O species and were thus out‐of‐line with most of the earlier spectroscopy. Photoreduction of metal ions in the synchrotron beam is now known to be an issue that affects oxidation states of metal ions in metalloprotein structures,^[6]^ including those for the early Compound I crystal structures (reviewed in refs. [1b, 7]). X‐ray free electron lasers (XFEL) avoid this problem because it becomes possible to collect the diffraction data within femtosecond timescales, which allows data collection before the X‐ray induced photoreduction/radiation damage take place.^[8]^

In this work, we present XFEL structures of Compound II in two different peroxidases—cytochrome *c* peroxidase and ascorbate peroxidase. The data indicate differences in the nature of the ferryl species between the Compound II intermediates in the two enzyme intermediates. The functional implications of this finding, in terms of proton delivery to the heme, are discussed.

## Results

**Compound II in C*c*P**. Of all the heme enzymes, C*c*P provides a very convenient framework for examination of ferryl reactivity. But while formation of Compound I in C*c*P is straightforward (because Compound I is a stable intermediate), formation of Compound II with high purity is not. This is in part because the UV‐visible spectra of Compounds I and II are very similar^[9]^ which makes it difficult to differentiate the two. And, it means that the most convenient and commonly used method for Compound II formation—namely by decay of Compound I (e.g.[^10^, ^11^])—cannot be used for C*c*P.

**Figure 1:**
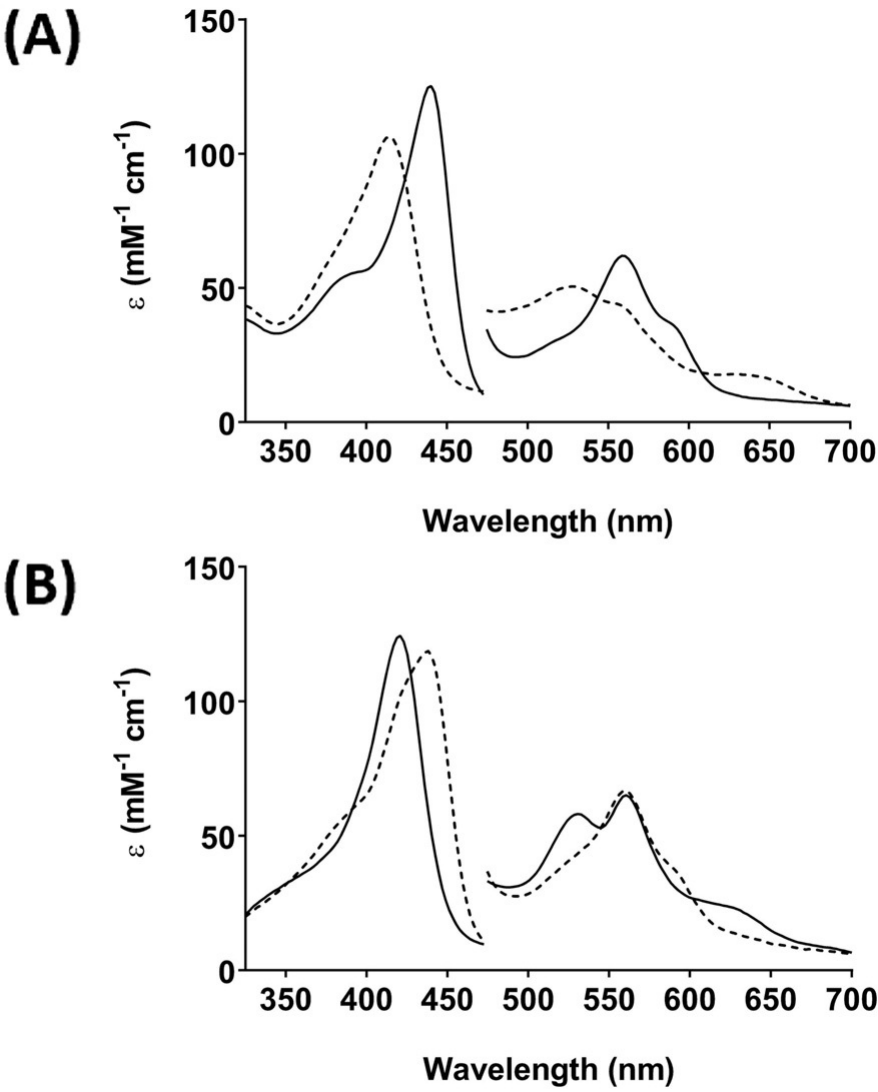
Formation of Compound II in C*c*P monitored by stopped‐flow. A) Formation of ferrous C*c*P: Ferric C*c*P (6 μM) was mixed with 2–3 equivalents of dithionite and spectra monitored over 10 s. The dashed line is the first species observed after mixing and represents the ferric enzyme, and the solid line represents the ferrous spectrum which was completely formed within 10 s of the mixing event. Absorbance values in the visible region have been multiplied by a factor of three. Conditions: 10 mM potassium phosphate, 150 mM KCl pH 6.5, 25.0 °C. This ferrous species was stable for at least 15 minutes at room temperature, after which time it decayed back to ferric. B) Formation of C*c*P Compound II: In a sequential mixing experiment ferric C*c*P (6 μM) was premixed with a stoichiometric amount of dithionite for 10 s (to enable complete formation of ferrous C*c*P) then H_2_O_2_ (5 equivalents) was added and spectral changes were monitored over 10 s. The dashed line is the first species observed after mixing and represents the ferrous species (at *t*=1 ms); this spectrum (*λ*_max_=439, 558, 590^sh^ nm) is similar to the spectrum of the ferrous enzyme formed in the single mix experiments in (A). The solid line after reaction with H_2_O_2_ is assigned as a Compound II species (*λ*_max_=420, 530, 560 nm). Over longer timescales (≈500 s), this Compound II species was observed to decay slowly (*k*_obs_≈0.004 s^−1^) back to a ferric‐like species (Figure S1).

**Figure 2:**
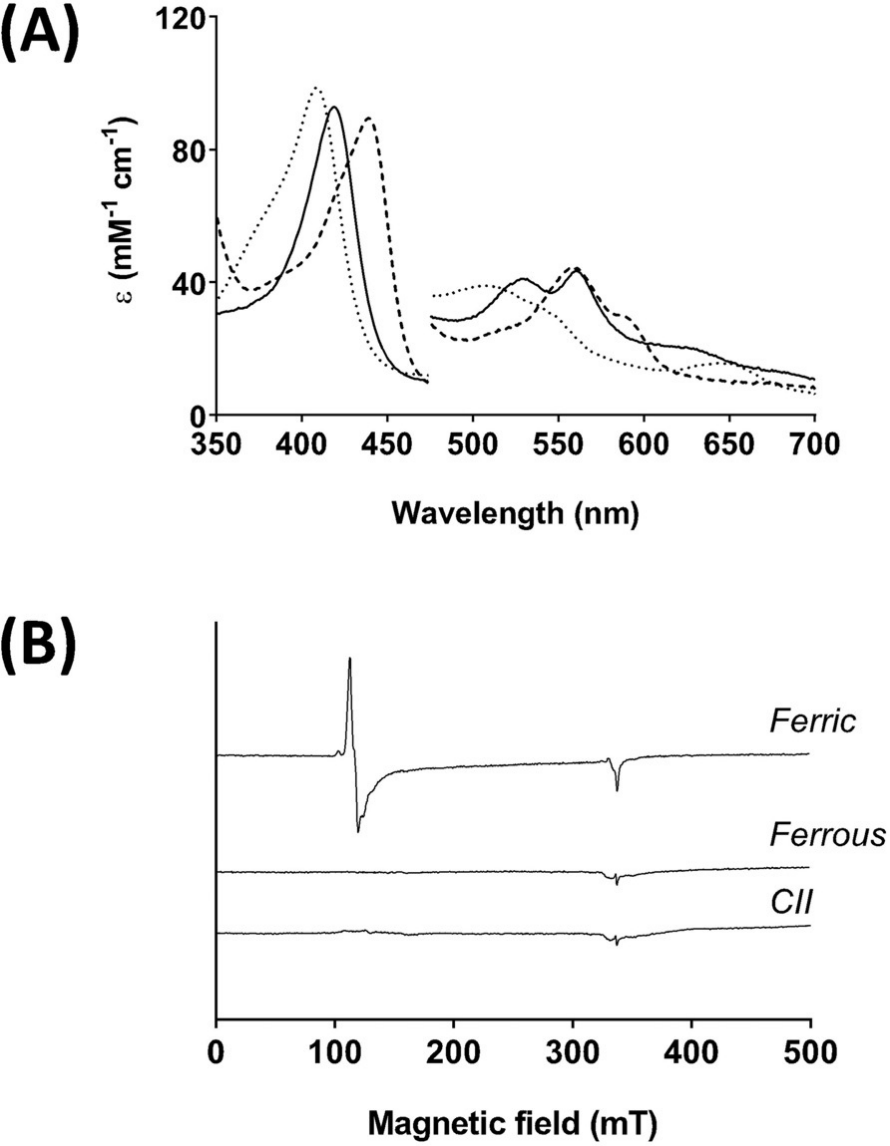
Formation of Compound II in C*c*P monitored by EPR. A) Absorption spectra of ferric, ferrous and Compound II forms of C*c*P used for EPR analysis: Ferric C*c*P (250 μM, dotted line) was reacted with 5–10 equivalents of dithionite to produce ferrous C*c*P (dashed line); Compound II (black solid line) was prepared by reaction of ferrous protein with 10 equivalents of H_2_O_2_. All spectra were recorded immediately after mixing and prior to flash freezing. Absorbance values in the visible region have been multiplied by a factor of three. Conditions: 10 mM potassium phosphate, 150 mM KCl pH 6.5, 25.0 °C. B) EPR spectra −9 GHz EPR spectra of the corresponding solutions from the experiments in (A) of the ferric (top spectrum), ferrous (middle spectrum) and Compound II (bottom spectrum) derivatives of C*c*P prepared by reaction of ferrous C*c*P with 10 equivalents of H_2_O_2_ and flash frozen immediately after mixing (see Experimental Section in the Supporting Information).

We thus developed an anaerobic procedure for direct formation of Compound II in C*c*P (see Experimental). Under anaerobic conditions, reaction of ferric C*c*P with dithionite led to formation of a stable ferrous species (*λ*_max_=440, 558, 590^sh^ nm, Figure 1 A). Further reaction with H_2_O_2_ yielded a species with wavelength maxima (*λ*_max_=420, 530, 560 nm, Figure 1 B) similar to those reported for Compound II of C*c*P.^[9]^ EPR was used to verify Compound II formation; Compound I is EPR‐active and Compound II is not. Absorption spectra of EPR samples were recorded prior to flash freezing in EPR tubes, to confirm the correct spectral features (Figure 2 A). The EPR signals characteristic of high‐spin ferric C*c*P (*g*⊥=6 and *g*_∥_=1.99) disappear in the ferrous and Compound II spectra, both of which are EPR‐silent, Figure 2 B. No new resonances are observed in the EPR‐silent spectrum of Compound II, consistent with the presence of a ferryl heme (*S=*1 species).

**Figure 3:**
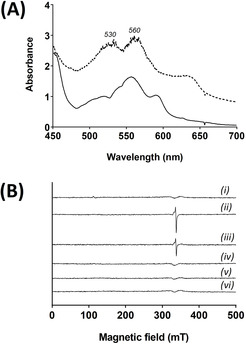
Formation of Compound II of C*c*P in crystals. A) Single crystal UV‐visible spectra (100 K) of crystals formed by reaction with dithionite (to give ferrous C*c*P, solid line), followed by reaction with 0.2 mM H_2_O_2_ (to give Compound II, dashed line). B) 9 GHz EPR spectra of single crystals of C*c*P. (i) Ferric C*c*P. (ii) Compound I formed by reaction of a ferric crystal with 0.2 mM H_2_O_2_. (iii) After storage of the crystal in (ii) for 20 days in liquid nitrogen; (iv) Compound II formed by reaction with dithionite and H_2_O_2_ as in (A). (v) After storage of the sample in (iv) for 20 days in liquid nitrogen; (vi) Background.

Single crystal spectrophotometry at 100 K on crystals of C*c*P that had been reduced with dithionite under anaerobic conditions, followed by anaerobic reaction with H_2_O_2_ (see Experimental), showed absorption peaks for Compound II (*λ*_max_=530, 560 nm, Figure 3 A) that reproduce those in solution above. Single crystal EPR spectra, Figure 3 B, also show formation of an EPR‐silent species (*i.e*. Compound II).

**Figure 4:**
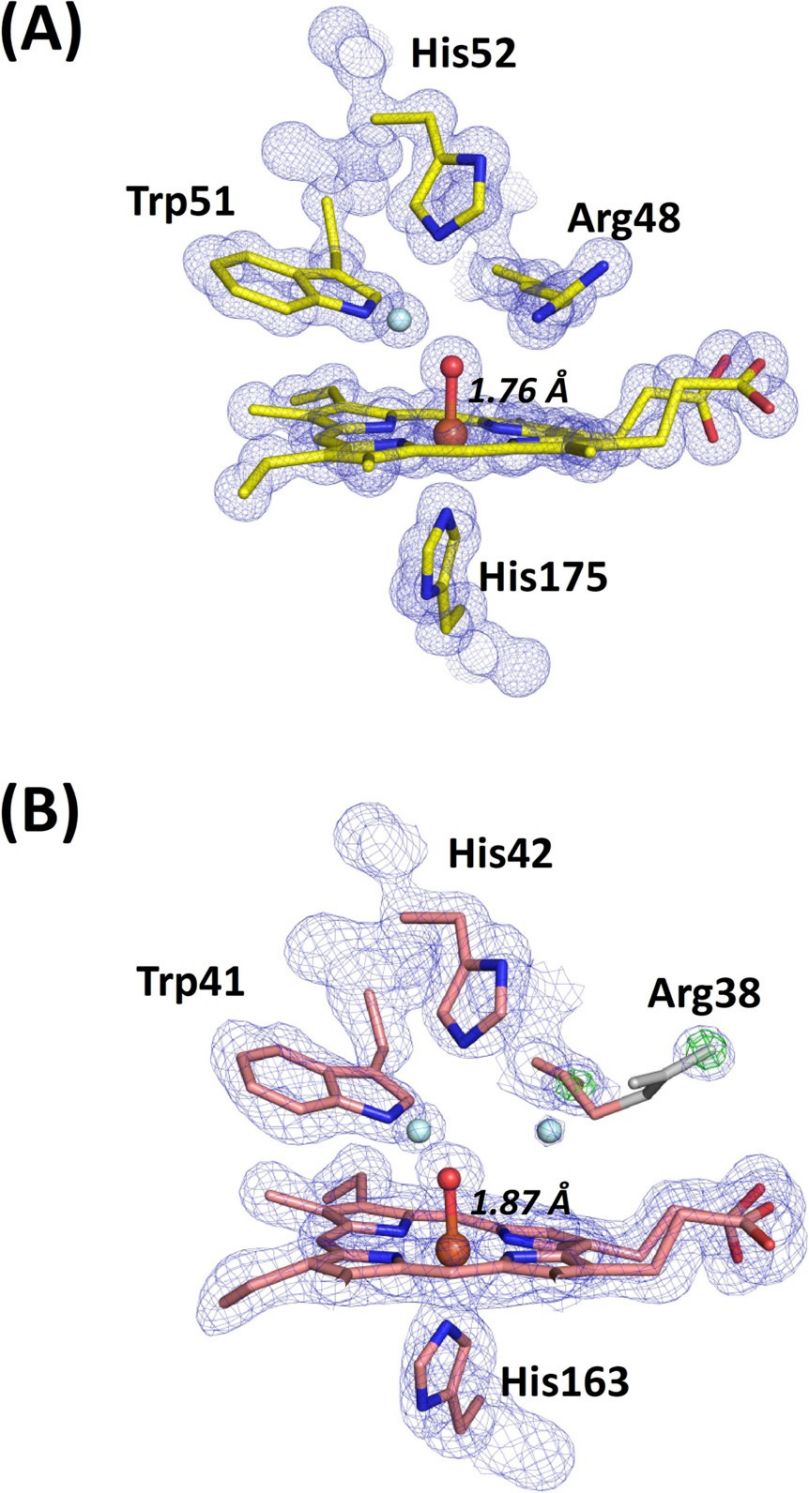
XFEL crystal structures of Compound II. A) C*c*P Compound II: Electron density of C*c*P Compound II is shown in blue (contoured at 2.0 σ). The O atom is positioned at 1.76 Å from the heme iron. Oxygen atoms of water molecules are shown in light blue, and the ferryl oxygen is shown in red (to differentiate it from water). B) APX Compound II: Electron density of APX Compound II is shown in blue (contoured at 2.0 σ). The difference density calculated by omitting Arg 38 is shown in green (contoured at 3 σ). Note the weak density beyond the Cδ of the side chain for Arg 38 (shown at 1.2 σ). Atoms of Arg38 are shown in grey where the atomic positions are unclearly defined. The ferryl O atom is positioned at 1.87 Å from the heme iron. Oxygen atoms of water molecules are shown in light blue, and the ferryl oxygen is shown in red, as for (A).

**Figure 5:**
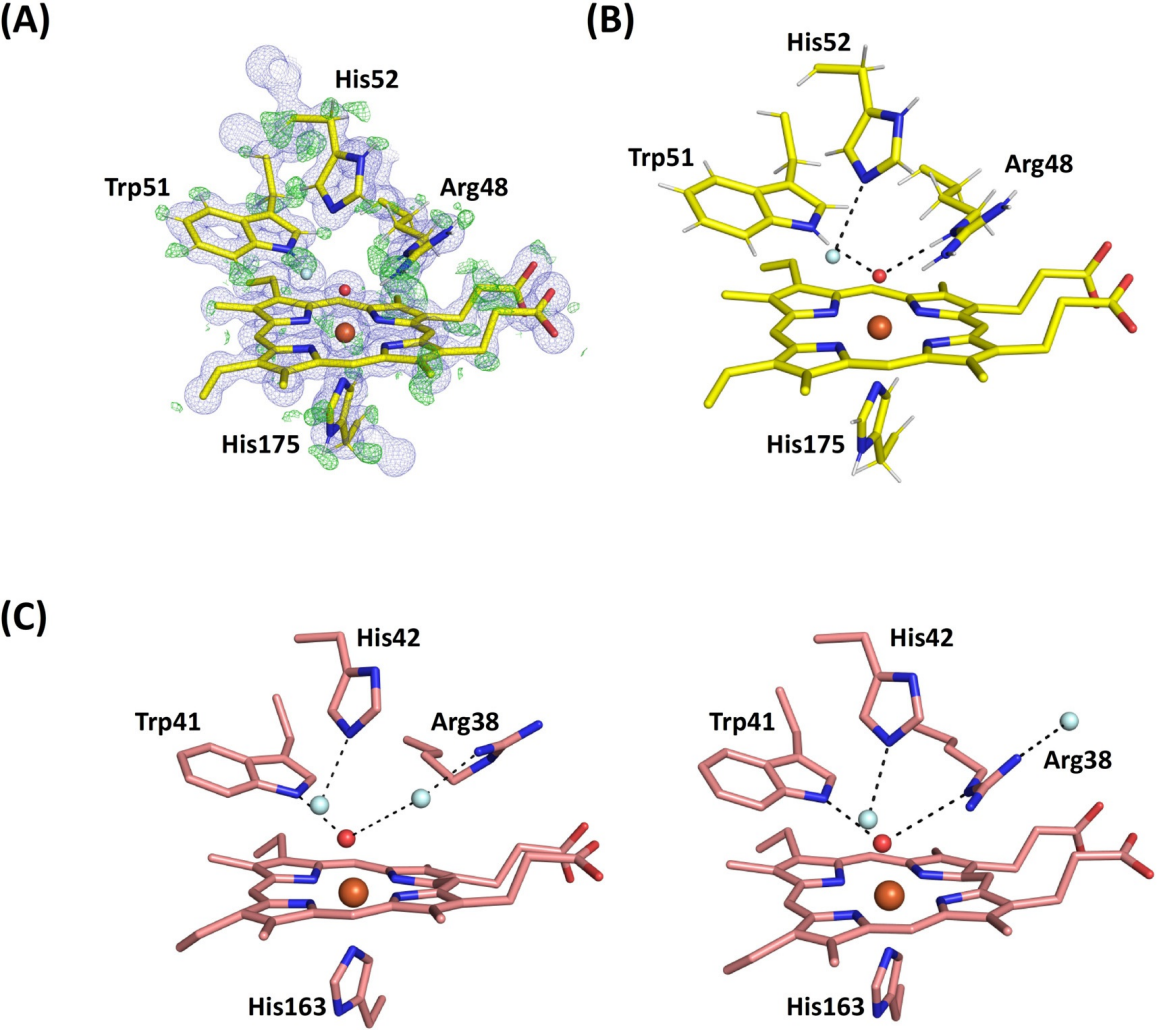
Hydrogen bonding in the active site. A) Electron density of C*c*P Compound II is shown in blue (contoured at 2.0σ). The difference density calculated by omitting hydrogens is shown in green (contoured at 3σ). The O atom is positioned at 1.76 Å from the heme iron. Water is shown in light blue color. B) Hydrogen bonding patterns for Compound II in C*c*P. C) Hydrogen bonding patterns for Compound II in APX showing Arg38 in two different locations: the “out” (left, as in Figure 4 B top) and “in” (right) positions. Waters are shown in light blue, and swap interchangeably as Arg38 moves between the two locations. Note that in the “out” position, Arg38 is hydrogen bonded to the water molecule through the Nη, and not as previously^[14]^ through the adjacent N*ϵ*. The ferryl oxygen is shown in red and hydrogen bonds are in dotted lines in all figures.

The X‐ray free electron laser (XFEL) structure of Compound II of C*c*P, prepared in crystals as above, was obtained at 1.06 Å resolution (PDB 7BIU).^[36]^ Data collection and refinement statistics are given in Table S1. An electron density map in the region of the heme pocket is shown in Figure 4 A. At this resolution it becomes possible to see the peaks in electron density associated with hydrogen atoms, and this difference density is shown in Figure 5 A. Note that although electron density at the positions of hydrogen atoms in Figure 5 A confirms their presence, absence of observed density cannot be taken as unequivocal proof of absence of a hydrogen at that point. Nonetheless, in this structure the N*ϵ* atom of His52 shows no density for a hydrogen atom and the imidazole is therefore more likely to be neutral (not protonated). This is in contrast to the neutron structure of Compound I in C*c*P,^[12]^ in which the N*ϵ* atom of His52 is carrying a proton. This change in protonation is consistent with a role for the distal histidine residue in regulating proton delivery to the ferryl species. Trp51 retains its hydrogen on N*ϵ*. The density for the guanidinium group of Arg48 is flat with all the hydrogens in‐plane, which is as expected when Arg48 is positively charged (Arg48 is also observed as charged in the neutron structure of Compound I^[12]^). At this resolution, we would expect to observe any deviation from planarity of the positively charged state that would arise if Arg48 was neutral.

The Fe−O bond length extracted from this XFEL structure of C*c*P Compound II is 1.76 Å (with an error estimate from SHELX^[13]^ of 0.013 Å). The complete models were refined with SHELXL^[13]^ allowing estimation of individual atomic positional uncertainties (ESU). The ESU of the iron and oxygen positions are 0.004 and 0.02 Å respectively. There are several key points to note when comparing with Compound I. First, the hydrogen bond patterns to the ferryl are the same in C*c*P Compound I and II (i.e. hydrogen bonds to the N*ϵ* atoms on Arg48 and the N*ϵ* proton of Trp 51, Figure 5 B) and do not allow space for a OH species bonded to the ferryl. These hydrogen bonds to Arg48 and Trp51 appear to be “locked in”, as the same hydrogen bonding pattern is observed in the X‐ray structure of C*c*P Compound I^[10]^ and in the neutron structure,^[12]^ Figure 5 B. Since the neutron structure of Compound I in C*c*P conclusively identifies a Fe^IV^=O species (from the absence of nuclear density for a OH species), then we therefore interpret this ferryl species in Compound II of C*c*P as also being unprotonated (i.e. a Fe^IV^=O species). This would be consistent with the fact that the uv‐visible spectra of Compounds I and II are indistinguishable. That said, the bond length of 1.76 Å in this C*c*P Compound II structure is measurably and (within error) longer than that for C*c*P Compound I (1.63 Å[^10^, ^12^]). Since this bond lengthening does not affect the hydrogen bonding patterns on the distal side, we take this to mean that the bond length of an unprotonated Fe^IV^=O species can be flexible within a single enzyme (and therefore that factors other than these distal hydrogen bonds affect bond length). We elaborate these ideas in the Discussion.

**Compound II in APX**. In a set of parallel experiments, single crystals of Compound II in APX were prepared by reaction with *m*‐chloroperbenzoic acid, as previously.^[14]^ Accordingly, an X‐ray free electron laser structure of Compound II of APX was also obtained at 1.50 Å resolution (PDB 7BI1, see Table S1).^[36]^ In this structure (unlike C*c*P above), the resolution is not high enough to see hydrogen atoms. An electron density map of the distal heme region is shown in Figure 4 B. In this structure, the iron‐oxygen bond length is 1.87 Å (with an error estimate from SHELX of 0.046 Å), and the ESU of the iron and oxygen positions are 0.014 and 0.083 Å respectively. This bond length in Compound II in APX is longer than that found in C*c*P and agrees with previous X‐ray^[10]^ and neutron^[14]^ structures. While bond lengths are determined from much lower resolution data in neutron experiments, the X‐ray,^[10]^ neutron^[14]^ and XFEL (this work) data are, nevertheless, in agreement with one another. Positive nuclear density has identified an Fe^IV^‐OH species for Compound II in APX,^[14]^ which is in agreement with the longer bond.

The electron density for the side‐chain of Arg38 (equivalent to Arg48 in C*c*P) is weak, occupying two different positions, Figure 5 C. In one location (the “in” position (14)) the N*ϵ* atom of Arg38 is hydrogen bonded to the ferryl heme, exactly as in C*c*P above. In the other position (“out”), Arg38 moves away from the ferryl group, and exchanges with a water molecule (Figure 5 C). This movement of the distal Arg residue is not evident in C*c*P. The functional implications of these differences between APX and C*c*P are discussed below.

## Discussion

A variety of methods have been used to examine the nature of ferryl heme (Fe^IV^=O versus Fe^IV^‐OH). These include resonance Raman and Mossbauer spectroscopies, EXAFS, and X‐ray crystallography. Many of these studies have been carried out on peroxidase enzymes, because their ferryl intermediates are well known and are more stable than the corresponding species in other enzymes (such as P450s) which use the same intermediates. It is worth remembering that none of the spectroscopic methods, nor X‐ray diffraction (unless at resolutions of ca. 1 Å, where the electrons of hydrogen atoms may be seen), can directly identify the locations of hydrogens so the positions of protons are inferred. To add to the technical difficulty, X‐ray induced photoreduction, particularly of positively charged centres such as metal ions is acknowledged to occur during crystal structure determination.^[15]^ Photoreduction can also be an issue in X‐ray absorption spectroscopy studies^[16]^ and resonance Raman studies.^[17]^

One method where hydrogens (usually as deuterons) are observed directly is in neutron diffraction, and in a neutron experiment photoreduction does not occur because the neutrons are scattered by nuclei and not (as in an X‐ray experiment) by electrons. We have previously used neutron diffraction to examine the ferryl species in Compound I of cytochrome *c* peroxidase. The nuclear density maps demonstrate that Compound I in C*c*P is an Fe^IV^=O species and is not protonated.^[12]^ These conclusions on Compound I agree with measurements of bond lengths in multi‐crystal X‐ray[^10^, ^18^] and XFEL^[19]^ studies on C*c*P, and with kinetic and spectroscopic work on Compound I in two proximal thiolate‐ligated heme enzymes (cytochrome P450^[20]^ and the fungal peroxygenase from *A. aegerita* ^[21]^). A consensus on Compound I therefore seems to be emerging.

The protonation state of the ferryl heme in Compound II is less well clarified. Positive nuclear density in the neutron crystal structure of Compound II of ascorbate peroxidase (APX)^[14]^ definitely identifies a OH ligand (Fe^IV^‐OH), although recent spectroscopic information is in favour of an unprotonated species.^[22]^ One philosophy^[11]^ is that histidine‐ligated heme systems are constitutionally unable to form a Fe^IV^‐OH species, because they lack a proximal thiolate (electron donating) ligand which is considered a key requirement. On the other hand, heme enzymes without a thiolate ligand can form Fe^IV^‐OH,^[23]^ so the presence of a thiolate ligand is not essential for Fe^IV^‐OH formation in all cases.

For Compound II, more information is needed to unpick these key biological questions. XFEL provides an alternative approach to both traditional X‐ray crystallography and to neutron crystallography techniques, in the sense that structures can be generated on femtosecond timecales that avoid X‐ray induced photoreduction or radiation damage. The XFEL structure of Compound II of C*c*P, resolved to 1.06 Å, shows a Fe−O bond length of 1.76 Å. This bond length is longer than that obtained using a multi‐crystal X‐ray method for Compound I of C*c*P (1.63 Å, determined at 1.67 Å resolution and with an ESU of the iron and oxygen atom positions of 0.017 and 0.066 Å respectively^[10]^). The corresponding XFEL structure of Compound II in APX, resolved at 1.50 Å, shows a Fe‐O distance of 1.87 Å which is almost identical to that obtained from multi‐crystal X‐ray (1.84 Å^[10]^) and neutron structures (1.88 Å,^[14]^ as shown in Figure S3).

### Comparison with Other Ferryl Species

Empirically‐determined distances for Fe−O bond lengths are quite reasonably often used as a binary determinant of bond order (single or double bond) and, by implication, of protonation state in ferryl complexes. But the comparison of the closely related C*c*P and APX enzymes above and the analysis below indicates that the situation might be considerably more complicated. Comparison of C*c*P and APX shows evident flexibility (or certainly variability) in the bond lengths of ferryl species. The most reliable bond lengths for Compound I in proteins have typically been within a range of *ca* 1.63–1.73 Å (Table S2) and are assigned as arising from Fe^IV^=O species. For Fe^IV^‐OH species, the bond is expected to be longer than for Fe^IV^=O, but by how much? The Fe^IV^=O bond length itself can vary, as we show above for Compounds I and II in C*c*P and also in Table S1, so there may be movement in these protein bond lengths under certain conditions. This would mean that there could be overlap between the expected bond lengths for Fe^IV^=O, Fe^IV^‐OH and even Fe^III^‐OH species, making them hard to differentiate by virtue of bond length alone.

There is evidence to support such flexibility in other enzymes. Consider for example the Fe^IV^‐OH bond length for chloroperoxidase Compound II (1.82 Å^[24]^) which is very close to the Fe^III^‐OH bond length in myoglobin (1.86 Å^[25]^). Consider also that in Compound II of *H. pylori* catalase^[23a]^ the Fe−O bond length (1.78 Å) has been interpreted^[23a]^ as a Fe^IV^‐OH species (in agreement with *P. mirabilis* catalase^[23b]^), but is arguably is within range of an unprotonated (Fe^IV^=O) species (e.g. 1.73 Å^[18]^). That the bond lengths for some Compound II intermediates are in a range that overlap with Fe^III^‐OH, and that bond lengths for Fe^IV^=O and Fe^IV^‐OH species can be closely merged together in different proteins, blurs the boundaries between individual species. We note also a recent XFEL analysis^[26]^ of a stable Compound I species in a dye‐decolorising peroxidase (DyP), which shows the expected short Fe−O bond length in one monomer of the crystal (1.65 Å), but much longer bonds (1.70–1.89 Å) in five others of the asymmetric unit at both room temperature (from XFEL data) and at 100 K (from synchrotron data).

**Figure 6:**
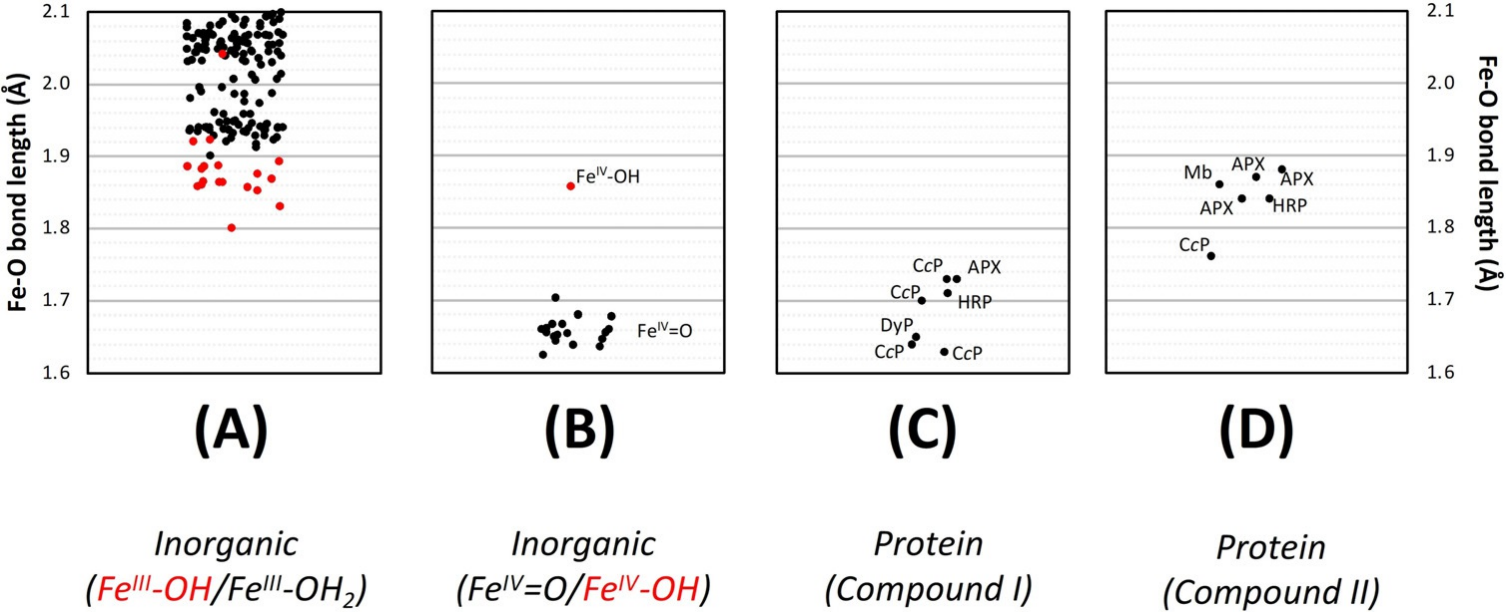
Comparison of Fe−O bond lengths obtained for various ferric (A) and ferryl (B) inorganic structures in the Cambridge Structure Database, alongside those for Compound I and Compound II protein structures (C, D). Photoreduction is not a complicating factor in small‐molecule crystal structures. A) Plot showing Fe−O bond lengths for complexes of iron in the ferric oxidation state in the Cambridge Structure Database. The plot shows only ferric structures with hydroxide (OH, highlighted in red circles) or water (H_2_O, black) as a ligand to the iron. Not shown in this plot, are structures for a Fe^III^=O species (Fe‐O=1.81 Å^[35]^). The majority (all but three) of the Fe^III^‐OH structures in the CSD lie in the range 1.8–1.9 Å. B) The equivalent plot for complexes of iron in the ferryl oxidation state, in the Cambridge Structure Database. All structures are assigned as Fe^IV^=O species, with the exception one structure which is assigned as Fe^IV^‐OH (red circle ^[27]^). Exact bonds lengths for each of the individual Fe^IV^ species are given in the SI. C) Fe−O bond lengths obtained from crystal structures of Compound I (see also Table S2). D) The same as (C), but for Compound II (see also Table S2).

An examination of structures of inorganic ferryl complexes further elaborates the above analysis. A range of bond lengths is observed for genuine Fe^IV^=O species (1.62–1.70 Å, Figure 6 B); these are notably shorter than the equivalent Fe^III^‐OH species, Figure 6 A. The range of Fe−O bond lengths for Compound I protein species (1.63–1.73 Å) is wider, but in reasonable agreement of those for the inorganic complexes (compare Figure 6 B,C). Regardless of whether they are assigned as Fe^IV^=O or Fe^IV^‐OH species, the bond lengths for Compounds II are all longer (1.76 Å‐ 1.88 Å, Figure 6 D). The only structure for an inorganic Fe^IV^‐OH species reports a similar bond length of 1.86 Å,^[27]^ highlighted in Figure 6 B, but the both the inorganic and protein Fe^IV^‐OH species are in a region that overlaps with the numerous Fe^III^‐OH structures, Figure 6 A.

### Functional Implications

Taking all of this information together, while considering at the same time the enzymatic requirements to do with the O−O bond cleavage event, it appears that there is a range of bond lengths that any particular species (Fe^IV^=O or Fe^IV^‐OH) might populate. Flexing of the bond and the bonding parameters might better reflect the range of dynamic motions that occur around the ferryl heme in different heme enzymes during catalytic turnover. This could be moderated by variables such as axial ligand, local charge, temperature, secondary coordination sphere and in particular hydrogen bonding^[28]^ as discussed below.

The data from the two XFEL structures of Compound II highlights differences in hydrogen bonding in the active site of these two closely related heme enzymes. In Compound II of APX, the distal Arg38 residue is mobile and is observed in two conformations in the structure. This is not observed in C*c*P. Movement of Arg38 changes the hydrogen bonding patterns in the active site, as shown in Figure 5 C. Arg38 is involved in proton delivery from the substrate (ascorbate) to the heme group during catalysis.^[29]^ And, neutron crystallography^[30]^ supports a role for Arg38 in proton delivery because Arg38 is neutral (having lost a proton in the complex) in the APX‐ascorbate complex. In one of the two locations for Arg38 (the “out” position, Figure 5 C (left)), there is a water molecule hydrogen bonded to the ferryl oxygen. In the other “in” position (Figure 5 C (right)), Arg38 hydrogen bonds to the ferryl oxygen in the same way as in C*c*P (Figure 5 B); in this conformation Arg38 would need to be a hydrogen acceptor to a proton on the ferryl oxygen, and this can only be achieved if Arg38 is in the neutral state (as seen in^[30]^). This highly dynamic hydrogen bonding arrangement in APX is presumably linked to the functional need for proton transfer from the substrate (ascorbate) and mediated by movement of Arg38—it involves two proton donors (water and Arg38) in two conformations. This could account for the longer Fe−O bond in Compound II of APX compared to C*c*P, and would provide a mechanism to facilitate movement of protons in an out of the active site. Hydrogen bonding to the distal histidine residue (or in other words whether the distal histidine is protonated or not) might also be important in regulating the bonding parameters.^[31]^ There is strong evidence from this study and our previous work for different protonation states of the distal histidine,[^12^, ^14^, ^30^] which will change the hydrogen bonding patterns. We note also that pH‐dependent changes in resonance Raman stretching frequencies for Compound II of both horseradish peroxidase^[32]^ and myoglobin^[25]^ are generally considered to arise from changes in hydrogen bonding to the ferryl.

By comparison, as we have shown above, no such flexibility in hydrogen bonding to the distal Arg is observed in C*c*P, as the active site hydrogen bond structure is “locked” in a fixed arrangement that does not change between Compounds I and II. That the electron transfer delivery pathway between ascorbate and the ferryl heme in APX (through Arg38 and the heme propionate^[33]^) is not replicated in C*c*P would be consistent with the different patterns of hydrogen bonding that we observe. In C*c*P, the electrons are delivered from cytochrome *c* through the proximal side,^[34]^ and so shuttling of protons from a substrate (at the heme edge) to the ferryl, mediated by the distal Arg, and changes in hydrogen bonding, are not a requisite part of the mechanism.

These results demonstrate not only the precise fine‐tuning that can exist within structurally almost identical heme active sites, but the ways in which this is connected to functional differences to do with substrate specificity. This information will feed into de novo design of new catalysts, aimed at mimicking heme protein reactivity and selectivity.

## Conflict of interest

The authors declare no conflict of interest.

## Supporting information

As a service to our authors and readers, this journal provides supporting information supplied by the authors. Such materials are peer reviewed and may be re‐organized for online delivery, but are not copy‐edited or typeset. Technical support issues arising from supporting information (other than missing files) should be addressed to the authors.

Supplementary

## References

[1a] J. T. Groves , J. Inorg. Biochem. 2006, 100, 434–447; 16516297 10.1016/j.jinorgbio.2006.01.012

[1b] P. C. E. Moody , E. L. Raven , Acc. Chem. Res. 2018, 51, 427–435. 29327921 10.1021/acs.accounts.7b00463

[2a] D. Keilin , T. Mann , Proc. R. Soc. London Ser. B 1937, 122, 119–133;

[2b] H. Theorell , Enzymologia 1941, 10, 250–252.

[3a] D. Keilin , E. F. Hartree , Biochem. J. 1951, 49, 88–104; 14848036 10.1042/bj0490088 PMC1197462

[3b] P. George , Nature 1952, 169, 612–613; 14929252 10.1038/169612a0

[3c] P. George , J. Biol. Chem. 1953, 201, 427–434. 13044812

[4a] J. T. Groves , Nat. Chem. 2014, 6, 89–91; 24451580 10.1038/nchem.1855 PMC3966521

[4b] S. G. Sligar , Science 2010, 330, 924–925; 21071657 10.1126/science.1197881 PMC4103181

[4c] K. D. Karlin , Nature 2010, 463, 168–169. 20075910 10.1038/463168a

[5] R. K. Behan , M. T. Green , J. Inorg. Biochem. 2006, 100, 448–459. 16500711 10.1016/j.jinorgbio.2005.12.019

[6a] J. Yano , J. Kern , K. D. Irrgang , M. J. Latimer , U. Bergmann , P. Glatzel , Y. Pushkar , J. Biesiadka , B. Loll , K. Sauer , J. Messinger , A. Zouni , V. K. Yachandra , Proc. Natl. Acad. Sci. USA 2005, 102, 12047–12052; 16103362 10.1073/pnas.0505207102 PMC1186027

[6b] T. Beitlich , K. Kuhnel , C. Schulze-Briese , R. L. Shoeman , I. Schlichting , J. Synchrotron Radiat. 2007, 14, 11–23; 17211068 10.1107/S0909049506049806

[6c] A. Ebrahim , T. Moreno-Chicano , M. V. Appleby , A. K. Chaplin , J. H. Beale , D. A. Sherrell , H. M. E. Duyvesteyn , S. Owada , K. Tono , H. Sugimoto , R. W. Strange , J. A. R. Worrall , D. Axford , R. L. Owen , M. A. Hough , IUCrJ 2019, 6, 543–551. 10.1107/S2052252519003956 PMC6608622 31316799

[7] T. L. Poulos , Chem. Rev. 2014, 114, 3919–3962. 24400737 10.1021/cr400415k PMC3981943

[8a] I. Inoue , Y. Inubushi , T. Sato , K. Tono , T. Katayama , T. Kameshima , K. Ogawa , T. Togashi , S. Owada , Y. Amemiya , T. Tanaka , T. Hara , M. Yabashi , Proc. Natl. Acad. Sci. USA 2016, 113, 1492–1497; 26811449 10.1073/pnas.1516426113 PMC4760814

[8b] K. Hirata , K. Shinzawa-Itoh , N. Yano , S. Takemura , K. Kato , M. Hatanaka , K. Muramoto , T. Kawahara , T. Tsukihara , E. Yamashita , K. Tono , G. Ueno , T. Hikima , H. Murakami , Y. Inubushi , M. Yabashi , T. Ishikawa , M. Yamamoto , T. Ogura , H. Sugimoto , J. R. Shen , S. Yoshikawa , H. Ago , Nat. Methods 2014, 11, 734–736; 24813624 10.1038/nmeth.2962

[8c] R. Neutze , R. Wouts , D. van der Spoel , E. Weckert , J. Hajdu , Nature 2000, 406, 752–757. 10963603 10.1038/35021099

[9] A. E. Pond , G. S. Bruce , A. M. English , M. Sono , J. H. Dawson , Inorg. Chim. Acta 1998, 275-276, 250–255.

[10] A. Gumiero , C. L. Metcalfe , A. R. Pearson , E. L. Raven , P. C. Moody , J. Biol. Chem. 2011, 286, 1260–1268. 21062738 10.1074/jbc.M110.183483 PMC3020733

[11] T. H. Yosca , J. Rittle , C. M. Krest , E. L. Onderko , A. Silakov , J. C. Calixto , R. K. Behan , M. T. Green , Science 2013, 342, 825–829. 24233717 10.1126/science.1244373 PMC4299822

[12] C. M. Casadei , A. Gumiero , C. L. Metcalfe , E. J. Murphy , J. Basran , M. G. Concilio , S. C. Teixeira , T. E. Schrader , A. J. Fielding , A. Ostermann , M. P. Blakeley , E. L. Raven , P. C. Moody , Science 2014, 345, 193–197. 25013070 10.1126/science.1254398

[13] G. M. Sheldrick , Acta Crystallogr. Sect. C 2015, 71, 3–8. 10.1107/S2053273314026370 PMC4283466 25537383

[14] H. Kwon , J. Basran , C. M. Casadei , A. J. Fielding , T. E. Schrader , A. Ostermann , J. M. Devos , P. Aller , M. P. Blakeley , P. C. E. Moody , E. L. Raven , Nat. Commun. 2016, 7, 13445. 27897163 10.1038/ncomms13445 PMC5141285

[15a] E. de la Mora , N. Coquelle , C. S. Bury , M. Rosenthal , J. M. Holton , I. Carmichael , E. F. Garman , M. Burghammer , J. P. Colletier , M. Weik , Proc. Natl. Acad. Sci. USA 2020, 117, 4142–4151; 32047034 10.1073/pnas.1821522117 PMC7049125

[15b] E. F. Garman , M. Weik , Methods Mol. Biol. 2017, 1607, 467–489; 28573586 10.1007/978-1-4939-7000-1_20

[15c] H. Kwon , O. Smith , E. L. Raven , P. C. Moody , Acta Crystallogr. Sect. D 2017, 73, 141–147. 10.1107/S2059798316016314 PMC5297917 28177310

[16] G. N. George , I. J. Pickering , M. J. Pushie , K. Nienaber , M. J. Hackett , I. Ascone , B. Hedman , K. O. Hodgson , J. B. Aitken , A. Levina , C. Glover , P. A. Lay , J. Synchrotron Radiat. 2012, 19, 875–886. 23093745 10.1107/S090904951203943X PMC3480274

[17] J. Terner , V. Palaniappan , A. Gold , R. Weiss , M. M. Fitzgerald , A. M. Sullivan , C. M. Hosten , J. Inorg. Biochem. 2006, 100, 480–501. 16513173 10.1016/j.jinorgbio.2006.01.008

[18] Y. T. Meharenna , T. Doukov , H. Li , S. M. Soltis , T. L. Poulos , Biochemistry 2010, 49, 2984–2986. 20230048 10.1021/bi100238r PMC3202969

[19] G. Chreifi , E. L. Baxter , T. Doukov , A. E. Cohen , S. E. McPhillips , J. Song , Y. T. Meharenna , S. M. Soltis , T. L. Poulos , Proc. Natl. Acad. Sci. USA 2016, 113, 1226–1231. 26787871 10.1073/pnas.1521664113 PMC4747753

[20] J. Rittle , M. T. Green , Science 2010, 330, 933–937. 21071661 10.1126/science.1193478

[21] X. Wang , S. Peter , M. Kinne , M. Hofrichter , J. T. Groves , J. Am. Chem. Soc. 2012, 134, 12897–12900. 22827262 10.1021/ja3049223 PMC3518585

[22] A. P. Ledray , C. M. Krest , T. H. Yosca , K. Mittra , M. T. Green , J. Am. Chem. Soc. 2020, 142, 20419—20425. 10.1021/jacs.0c09108 PMC8107191 33170000

[23a] T. H. Yosca , M. C. Langston , C. M. Krest , E. L. Onderko , T. L. Grove , J. Livada , M. T. Green , J. Am. Chem. Soc. 2016, 138, 16016–16023; 27960340 10.1021/jacs.6b09693 PMC5987761

[23b] O. Horner , J. M. Mouesca , P. L. Solari , M. Orio , J. L. Oddou , P. Bonville , H. M. Jouve , J. Biol. Inorg. Chem. 2007, 12, 509–525. 17237942 10.1007/s00775-006-0203-9

[24] K. L. Stone , R. K. Behan , M. T. Green , Proc. Natl. Acad. Sci. USA 2006, 103, 12307–12310. 16895990 10.1073/pnas.0603159103 PMC1567876

[25] T. H. Yosca , R. K. Behan , C. M. Krest , E. L. Onderko , M. C. Langston , M. T. Green , J. Am. Chem. Soc. 2014, 136, 9124–9131. 24875119 10.1021/ja503588n PMC4091272

[26] M. Lučić , D. A. Svistunenko , M. T. Wilson , A. K. Chaplin , B. Davy , A. Ebrahim , D. Axford , T. Tosha , H. Sugimoto , S. Owada , F. S. N. Dworkowski , I. Tews , R. L. Owen , M. A. Hough , J. A. R. Worrall , Angew. Chem. Int. Ed. 2020, 59, 21656–21662; 10.1002/anie.202008622 PMC7756461 32780931

[27] J. P. T. Zaragoza , T. H. Yosca , M. A. Siegler , P. Moenne-Loccoz , M. T. Green , D. P. Goldberg , J. Am. Chem. Soc. 2017, 139, 13640–13643. 28930448 10.1021/jacs.7b07979 PMC6058725

[28a] V. F. Oswald , J. L. Lee , S. Biswas , A. C. Weitz , K. Mittra , R. Fan , J. Li , J. Zhao , M. Y. Hu , E. E. Alp , E. L. Bominaar , Y. Guo , M. T. Green , M. P. Hendrich , A. S. Borovik , J. Am. Chem. Soc. 2020, 142, 11804–11817; 32489096 10.1021/jacs.0c03085 PMC7899053

[28b] Z. Gordon , M. J. Drummond , E. M. Matson , J. A. Bogart , E. J. Schelter , R. L. Lord , A. R. Fout , Inorg. Chem. 2017, 56, 4852–4863. 28394119 10.1021/acs.inorgchem.6b03071

[29] I. Efimov , S. K. Badyal , C. L. Metcalfe , I. Macdonald , A. Gumiero , E. L. Raven , P. C. Moody , J. Am. Chem. Soc. 2011, 133, 15376–15383. 21819069 10.1021/ja2007017

[30] H. Kwon , J. Basran , J. M. Devos , R. Suardiaz , M. W. van der Kamp , A. J. Mulholland , T. E. Schrader , A. Ostermann , M. P. Blakeley , P. C. E. Moody , E. L. Raven , Proc. Natl. Acad. Sci. USA 2020, 117, 6484–6490. 32152099 10.1073/pnas.1918936117 PMC7104402

[31] K. Nilsson , H. P. Hersleth , T. H. Rod , K. K. Andersson , U. Ryde , Biophys. J. 2004, 87, 3437–3447. 15339813 10.1529/biophysj.104.041590 PMC1304810

[32] A. J. Sitter , C. M. Reczek , J. Terner , J. Biol. Chem. 1985, 260, 7515–7522. 3997887

[33] K. H. Sharp , M. Mewies , P. C. Moody , E. L. Raven , Nat. Struct. Biol. 2003, 10, 303–307. 12640445 10.1038/nsb913

[34] H. Pelletier , J. Kraut , Science 1992, 258, 1748–1755. 1334573 10.1126/science.1334573

[35a] C. E. MacBeth , A. P. Golombek , V. G. Young, Jr. , C. Yang , K. Kuczera , M. P. Hendrich , A. S. Borovik , Science 2000, 289, 938–941; 10937994 10.1126/science.289.5481.938

[35b] C. L. Ford , Y. J. Park , E. M. Matson , Z. Gordon , A. R. Fout , Science 2016, 354, 741–743; 27846604 10.1126/science.aah6886

[35c] E. M. Matson , Y. J. Park , A. R. Fout , J. Am. Chem. Soc. 2014, 136, 17398–17401. 25470029 10.1021/ja510615p

[36] Atomic coordinates have been deposited in the Protein Data Bank (PDB ID codes: 7BIU for C*c*P Compound II and 7BI1 for APX Compound II). Raw image data have been deposited with the Zenodo depository (DOI: 10.5281/zenodo.4484116 for 7BIU and DOI: 10.5281/zenodo.4484116 for 7BI1).

