# Supplementary material for: XFEL Crystal Structures of Peroxidase Compound II
Source: Angew Chem Weinheim Bergstr Ger. 2021 May 19;133(26):14699–706. doi: 10.1002/ange.202103010 (PMC10947387; doi:10.1002/ange.202103010)
Supplement: Supplementary file 1 — Supplementary [file ANGE-133-14699-s001.pdf]

Angewandte Chemie
Eine Zeitschrift der Gesellschaft Deutscher Chemiker

Supporting Information

XFEL Crystal Structures of Peroxidase Compound II

Hanna Kwon, Jaswir Basran, Chinar Pathak, Mahdi Hussain, Samuel L. Freeman, Alistair J. Fielding, Anna J. Bailey, Natalia Stefanou, Hazel A. Sparkes, Takehiko Tosha, Keitaro Yamashita, Kunio Hirata, Hironori Murakami, Go Ueno, Hideo Ago, Kensuke Tono, Masaki Yamamoto, Hitomi Sawai, Yoshitsugu Shiro, Hiroshi Sugimoto, Emma L. Raven, and Peter C. E. Moody**

ange_202103010_sm_miscellaneous_information.pdf

EXPERIMENTAL

Protein expression, purification and crystallization. Yeast cytochrome *c* peroxidase, in a pLEICS-03 vector carrying kanamycin resistance and a TEV-cleavable N-terminal His tag, was expressed in *E. coli* BL21 (DE3) cells. Cells were grown in 2-YT media at 37 °C until they reached an OD_{600} = 0.8, at which point they were induced with 1 mM IPTG and incubated at 22 °C overnight. Cells were harvested by centrifugation (4,000 x g at 277 K for 20 min) and protein purified as previously described [1]. Protein concentrations were determined from the reported[2] absorption coefficient (ε_{408} = 102 mM^{-1} cm^{-1}). The protein was crystallised at pH 6.0 according to published methods[1].

Soybean cytosolic APX in a pLEICS-03 vector carrying kanamycin resistance and a TEV cleavable N-terminal His tag was expressed in *E.coli* BL21 (DE3) cells. Protein was expressed in 2 x YT media for 16 h at 37 °C without induction with IPTG. Protein was purified as previously described[3]. Crystals were grown by vapour diffusion hanging drops made up of 2–4 µl protein (20 $mgml^{-1}$ in 10 mM potassium phosphate pH 7.0, 150 mM KCl) and an equal volume of precipitant (2.25 M Li_2SO_4, 0.1 M HEPES pH 8.3–8.9). The drop was allowed to equilibrate with 700 µl of precipitant. The crystals appeared 2–14 days at room temperature.

Stopped-flow kinetics. Pre-steady state stopped-flow experiments were carried out using an Applied Photophysics SX.20MV stopped-flow spectrometer housed in an anaerobic glove box (Belle Technology Ltd., [O_2] < 5 ppm). All experiments were carried out at 25.0 °C unless otherwise stated, using 10 mM potassium phosphate buffer, 150 mM KCl, pH 6.5. Spectral deconvolution was performed by global analysis and numerical integration methods using Pro-Kineticist software (Applied Photophysics Ltd).

Formation of Compound II. In peroxidases, Compound II is often generated by reaction of the ferric enzyme with peroxide; this leads to Compound I formation, which in all peroxidases will decay on different timescales to Compound II. In cases where Compound I is very unstable (such as in APX[4]), formation of Compound II is rapid and a stable species is obtained. Peroxybenzoic acids (*e.g. m*-chloroperbenzoic acid) are sometimes used instead of peroxide as this also leads to Compound I (and Compound II) formation, but avoids the complications associated with excess H_2O_2 which can lead to formation of a dead-end Compound III. In most peroxidases (such as APX), these methods are appropriate because the spectra of the Compound I (usually a ferryl heme with a porphyrin π-cation radical) and Compound II (ferryl only) species are different. But this is not the case in C*c*P, as the spectra of Compound I and II are almost identical[5]. Cognisant of previous methodologies[5-6], we thus developed an anaerobic procedure for reliable formation of Compound II of C*c*P directly from the ferrous enzyme.

In solution, Compound II of C*c*P was formed from the ferrous enzyme by reaction with H_2O_2. In stopped flow experiments, formation of ferrous C*c*P was monitored in single mixing mode by mixing ferric enzyme (typically 2-5 µM) with 2-3 equivalents of dithionite; this was sufficient to completely reduce ferric C*c*P to ferrous as shown in equilibrium titration experiments (see Fig. S2).

Time dependent spectral changes were monitored using a photodiode array detector. Data were fitted to a one-step model A → B, where A = ferric enzyme and B = ferrous enzyme. Compound II was generated from the ferrous enzyme by reaction of peroxide with the ferrous form of the enzyme using the sequential mixing mode of the stopped-flow instrument. The experiment was initiated by mixing ferric enzyme with a stoichiometric amount of sodium dithionite and then aging the solution for 10 s, to ensure complete formation of ferrous C*c*P before a second mix with five equivalents of H_2O_2. Time dependent spectral changes accompanying Compound II formation were followed and data analysis carried out as outlined above, in this case fitting to a one-step model A → B, where A = ferrous enzyme and B = Compound II.

In crystals, Compound II of C*c*P were obtained under anaerobic conditions (by handling in a glove box) by soaking crystals in dithionite for 1 min followed by soaking in 0.2 mM H_2O_2 for 1 min then flash frozen in liquid nitrogen. Compound II in single crystals of APX were prepared as previously[7] by reacting with *m*-CPBA (0.2 mM) for 40 s at 4 °C then flash frozen in liquid nitrogen.

Single crystal spectrophotometry. Single crystal spectra were collected using an Ocean Optics Maya 2000 PRO spectrometer, with an Ocean Optics DH-2000-BAL UV-VIS-NIR light source and a Hamamatsu S10420 FFT-CCD back-thinned detector with fibre optic coupled to 80 mm diameter 4X reflective lenses (Optic Peter, Lentilly, France) and mounted with a custom mount (BioMedical Mechanical Workshop, University of Leicester) on a Rigaku Raxis IV φ drive. Crystals were mounted on a nylon loop in a SPINE standard mount. The temperature was maintained at 100 K with an Oxford

Cryosystems cryostream. Absorption spectra were acquired by means of the Ocean Optics SpectraSuite software. 4-6 crystals from each 16-crystal puck were checked for Compound II formation before shipping to SACLA.

EPR Spectroscopy. Continuous-wave EPR spectra were recorded at 9.4 GHz on a Bruker EMX spectrometer with a Super-high-Q rectangular cavity and an Oxford ESR-900 liquid helium cryostat. The buffer used was 10 mM potassium phosphate buffer, 150 mM KCl, pH 6.5. All samples for EPR spectroscopy were prepared in an anaerobic glove box (Belle Technology Ltd) and their absorption spectra recorded using a Jasco V630 spectrophotometer (also housed within the glove box) prior to sample freezing, to confirm the correct spectral features. Compound II was prepared by manually mixing ferrous C*c*P (250 μM, prepared by the addition of 5-10 equivalents of dithionite to ferric C*c*P) with 10 equivalents of H_2O_2 solution directly in 4 mm quartz EPR tubes, followed by flash freezing in liquid nitrogen. Deuterated C*c*P crystals were reacted in the same way as for the single crystal spectrophotometry above. All spectra were recorded at 5 K, 0.1 mT modulation amplitude, 100 kHz, 1 mW, 2 scans, 2048 points.

XFEL data collection and structure determination. X-ray diffraction data of C*c*P and APX were collected at 100 K by the Serial Femtosecond Rotation Crystallography (SF-ROX) method[8] at BL2 EH3 of SACLA and using a pulse length <10 fs. The crystals were rotated with angle steps of 0.15° with a 50 μm shift between each shot to record the still diffraction images using a MX300-HS (Rayonix) CCD detector. Diffraction data for C*c*P were collected using an X-ray photon energy of 15.2 keV with a camera distance of 143.4 mm. The X-ray beam size at the sample point was 3.0 μm (horizontal) x 4.1 μm (vertical). The beam intensity was attenuated to ~34% (to 7.7 x 10^{10} photons per pulse) using a 0.5 mm Si plate to avoid detector saturation. Diffraction data for APX were collected using an X-ray photon energy of 10.0 keV with a camera distance of 103.0 mm. The X-ray beam size at the sample point was 2.8 μm (horizontal) x 5.1 μm (vertical). The X-ray beam was attenuated to ~22% (to 8.7 x 10^{10} photons per pulse) by 0.2 mm Si plate. The SPINE-standard mount with the crystal was placed on the goniometer using the automatic crystal exchange system SPACE. The crystals were kept at 100 K during data collection. The hit images were found using the program Cheetah[9]. The diffraction images were processed using the CrystFEL suite[10]. Indexing was carried out with DirAx[11]. Integrated intensities were merged by process_hkl in CrystFEL[10]. In the processing of C*c*P, version 0.9.1 was used and indexing was carried out with DirAx[11], Mosflm[12] and XGANDALF[13], followed by merging using process_hkl with options --scale and --push-res=3.7. In the processing of APX, version 0.6.3 was used and indexing was carried out with DirAx[11] and Mosflm[12], and then merging without scaling was performed. In the crystallographic refinement, manual model rebuilding was performed by COOT [14] and the model was subsequently refined by Phenix[15] and REFMAC[16]. Final refinement and ESU calculations were undertaken with SHELXL[17] with the Fe-O distance error estimates reported using the BOND keyword. Atomic coordinates have been deposited in the Protein Data Bank (PDB ID codes: 7BIU for C*c*P Compound II and 7BI1 for APX Compound II). In the case of APX CII, the refinement of isotropic B values increased the crystallographic R value to 0.20 without affecting the R_{free}.

Cambridge Structural Database (CSD) Search. ConQuest from the CCDC software (version 2020.2) was used to design a search of the CSD [18]. A combination query was designed to include Fe-O bonds but excluded structures where the oxygen atom was additionally bonded to any atom other than hydrogen. In addition, 3D parameters were set to constrain the Fe-O distance to between 1.5 Å to 2.1 Å. The iron redox state was manually entered for each data point, and structures with unclear redox states were discarded. A univariate scatter plot was produced and the data were overlayed with the Fe-O bond lengths of heme proteins (see Table 2). The Fe^{IV}-O distances (Å) obtained from the search and ordered from longest to shortest distance are: 1.857[19], 1.703[20], 1.68[21], 1.677[22], 1.667[23], 1.667[24], 1.661[25], 1.660[26], 1.660[27], 1.658[28], 1.656[22], 1.656[29], 1.654[29], 1.652[23], 1.650[23], 1.646[30], 1.644[23], 1.638[31], 1.636[23], 1.624[32].

Table S1. X-ray data collection and structure refinement.

Data name	C*c*P Compound II	APX Compound II
**Data collection**	2020-Jul-2	2018-Apr-3
Wavelength (Å)	0.816	1.24
Resolution (Å)	13.0-1.06	58-1.50
Highest shell (Å)	1.07-1.06	1.51-1.50
Space group	$P2_12_12_1$	$P4_22_12$
Cell dimensions (Å)	50.81, 75.54, 106.57	81.94, 81.94, 75.05
No. of crystals	147	147
No. of images (indexed/hit)	9145 / 10303	8747/8904
Measured reflections	70096226	27489038
Unique reflections	185757	41508
Completeness (%)	100.0 (100.0)	100.0 (100.0)
Redundancy	377.3 (174.0)	662.3 (449.5)
$CC_{1/2}$ (%)	0.970 (0.259)	0.988 (0.869)
R-split (%)	11.12 (50.59)	8.27 (26.67)
$\langle I/\sigma(I)\rangle$	6.34 (2.41)	11.2 (5.2)
**Refinement**		
R_{work}/R_{free} (%)	14/17	15/24
RMSD bond length (Å)	0.01	0.008
RMSD bond angle (°)	2.06	1.81

Table S2. Summary of most recent Fe-O bonds lengths measured by crystallography[a] or EXAFS.

Protein	Compound I (Å)	Compound II (Å)	Resolution (Å)	Interpretation	Ref
Dye-decolourising peroxidase	1.65[b]		1.8	Fe(IV)=O	[33]
Cytochrome *c* peroxidase	1.64[c]		2.2	Fe(IV)=O	[34]
	1.63[d]		1.7	Fe(IV)=O	[1]
	1.73[d]		1.4	Fe(IV)=O	[35]
	1.70[b]		1.5	Fe(IV)=O	[36]
		1.76[b,g]	1.0	Fe(IV)=O	This work
Ascorbate peroxidase	1.73[d]		1.7	Fe(IV)=O	[1]
		1.88[c]	1.8	Fe(IV)-OH	[7]
		1.84[d]	1.7	Fe(IV)-OH	[1]
		1.87[b]	1.5	Fe(IV)-OH	This work
Myoglobin		1.86[f]	1.3	Fe(IV)-OH	[37]
		1.66[e]		Fe(IV)-OH	[38]
Horseradish peroxidase	1.71[f]		1.6	Fe(IV)=O	[39]
		1.84[f]	1.6	Fe(IV)-OH	[39]
Chloroperoxidase	1.65[e]		[d]	Fe(IV)=O	[40]
		1.82[e]	[e]	Fe(IV)-OH	[41]
Cytochrome P450		1.82[e]	[e]	Fe(IV)-OH	[42]
H. pylori catalase	1.66[e]		[e]	Fe(IV)=O	[43]
		1.78[e]	[e]	Fe(IV)-OH	[43]

[a] Earlier crystal structures where photoreduction is a concern are not included in this table (*i.e.* 1.87 Å for C*c*P Compound I[44], 1.87 Å for catalase Compound II [45] and 1.92 Å for myoglobin Compound II[46]).
[b] XFEL.
[c] Neutron crystallography.
[d] Multi-crystal X-ray crystallography.
[e] EXAFS.
[f] Single crystal X-ray crystallography.
[g] The much higher resolution data means this distance is more reliable than (and therefore supersedes) previous X-ray measurements of C*c*P Compound II[1].

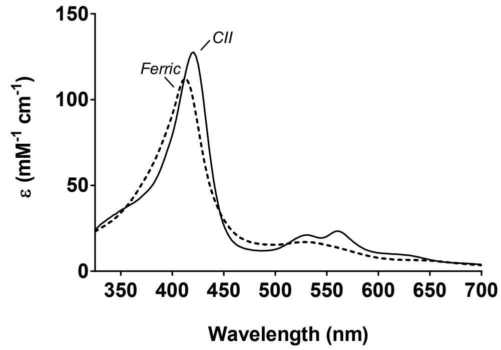

Figure S1. Decay of Compound II (solid line) back to ferric C*c*P (dashed line) over the course of 500 s. Absorbance values in the visible region have been multiplied by a factor of three. Conditions: 10 mM potassium phosphate, 150 mM KCl pH 6.5, 25.0 °C.

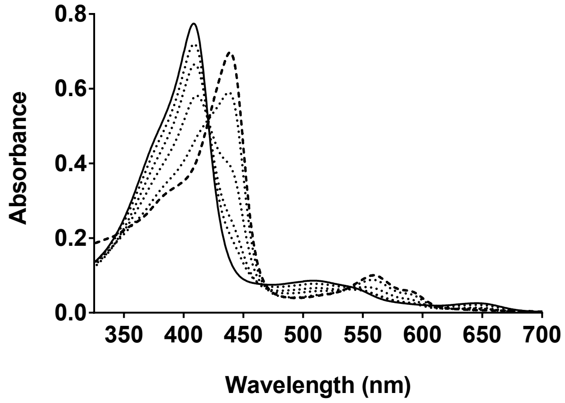

Figure S2. Formation of ferrous C*c*P under anaerobic conditions. Ferric C*c*P (7.5 μM) was titrated with one microlitre additions of a solution of dithionite (stock concentration = 2.1 mM) and the spectrum recorded after each addition. The solid line represents the ferric enzyme, and the dashed line represents the ferrous enzyme; dotted lines represent spectra recorded after sequential additions of dithionite. Dithionite was added until there was no further change in the absorbance at 438 nm (Soret band for ferrous enzyme), equivalent to a final dithionite concentration in the cuvette of 13 μM. Conditions: 10 mM potassium phosphate, 150 mM KCl pH 6.5, 25.0 °C. Experiments were carried out using a Jasco V630 UV-VIS spectrophotometer housed in a Belle Technology glove box.

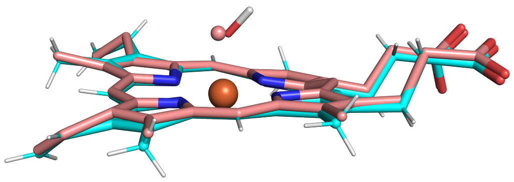

Figure S3. Overlay of the APX Compound II neutron structure[7], shown in cyan, and the XFEL structure obtained in this work, shown in pink. The distance between the OH and the heme iron is 1.88 Å for the neutron structure.

REFERENCES

[1] A. Gumiero, C. L. Metcalfe, A. R. Pearson, E. L. Raven, P. C. Moody, *J. Biol. Chem.* **2011**, *286*, 1260-1268.

[2] L. A. Fishel, J. E. Villafranca, J. M. Mauro, J. Kraut, *Biochemistry* **1987**, *26*, 351-360.

[3] a) I. K. Macdonald, S. K. Badyal, L. Ghamsari, P. C. Moody, E. L. Raven, *Biochemistry* **2006**, *45*, 7808-7817; b) K. H. Sharp, M. Mewies, P. C. Moody, E. L. Raven, *Nat. Struct. Mol. Biol.* **2003**, *10*, 303-307.

[4] W. R. Patterson, T. L. Poulos, D. B. Goodin, *Biochemistry* **1995**, *34*, 4342-4345.

[5] A. E. Pond, G. S. Bruce, A. M. English, M. Sono, J. H. Dawson, *Inorg Chim Acta* **1998**, *276*, 250-255.

[6] a) B. Ward, C. K. Chang, *Photochem Photobiol* **1982**, *35*, 757-759; b) P. S. Ho, B. M. Hoffman, C. H. Kang, E. Margoliash, *J Biol Chem* **1983**, *258*, 4356-4363; c) A. F. Coulson, J. E. Erman, T. Yonetani, *J Biol Chem* **1971**, *246*, 917-924.

[7] H. Kwon, J. Basran, C. M. Casadei, A. J. Fielding, T. E. Schrader, A. Ostermann, J. M. Devos, P. Aller, M. P. Blakeley, P. C. E. Moody, E. L. Raven, *Nat Commun* **2016**, *7*, DOI: 10.1038/ncomms13445.

[8] K. Hirata, K. Shinzawa-Itoh, N. Yano, S. Takemura, K. Kato, M. Hatanaka, K. Muramoto, T. Kawahara, T. Tsukihara, E. Yamashita, K. Tono, G. Ueno, T. Hikima, H. Murakami, Y. Inubushi, M. Yabashi, T. Ishikawa, M. Yamamoto, T. Ogura, H. Sugimoto, J. R. Shen, S. Yoshikawa, H. Ago, *Nature methods* **2014**, *11*, 734-736.

[9] A. Barty, R. A. Kirian, F. R. Maia, M. Hantke, C. H. Yoon, T. A. White, H. Chapman, *Journal of applied crystallography* **2014**, *47*, 1118-1131.

[10] T. A. White, V. Mariani, W. Brehm, O. Yefanov, A. Barty, K. R. Beyerlein, F. Chervinskii, L. Galli, C. Gati, T. Nakane, A. Tolstikova, K. Yamashita, C. H. Yoon, K. Diederichs, H. N. Chapman, *Journal of applied crystallography* **2016**, *49*, 680-689.

[11] A. J. M. Duisenberg, *J. Appl. Crystallogr.* **1992**, *25*, 92-96.

[12] A. G. W. Leslie, H. R. Powell, in *Evolving Methods for Macromolecular Crystallography, Vol. 245* (Eds.: R. J. Read, J. L. Sussman), Springer Netherlands, **2007**, pp. 41-51.

[13] Y. Gevorkov, O. Yefanov, A. Barty, T. A. White, V. Mariani, W. Brehm, A. Tolstikova, R. R. Grigat, H. N. Chapman, *Acta Crystallogr A Found Adv* **2019**, *75*, 694-704.

[14] P. Emsley, B. Lohkamp, W. G. Scott, K. Cowtan, *Acta Crystallographica Section D* **2010**, *66*, 486-501.

[15] D. Liebschner, P. V. Afonine, M. L. Baker, G. Bunkoczi, V. B. Chen, T. I. Croll, B. Hintze, L. W. Hung, S. Jain, A. J. McCoy, N. W. Moriarty, R. D. Oeffner, B. K. Poon, M. G. Prisant, R. J. Read, J. S. Richardson, D. C. Richardson, M. D. Sammito, O. V. Sobolev, D. H. Stockwell, T. C. Terwilliger, A. G. Urzhumtsev, L. L. Videau, C. J. Williams, P. D. Adams, *Acta Crystallogr D Struct Biol* **2019**, *75*, 861-877.

[16] G. N. Murshudov, P. Skubak, A. A. Lebedev, N. S. Pannu, R. A. Steiner, R. A. Nicholls, M. D. Winn, F. Long, A. A. Vagin, *Acta Crystallogr D Biol Crystallogr* **2011**, *67*, 355-367.

[17] G. M. Sheldrick, *Acta Crystallogr C Struct Chem* **2015**, *71*, 3-8.

[18] I. J. Bruno, J. C. Cole, P. R. Edgington, M. Kessler, C. F. Macrae, P. McCabe, J. Pearson, R. Taylor, *Acta Crystallogr B* **2002**, *58*, 389-397.

[19] J. P. T. Zaragoza, T. H. Yosca, M. A. Siegler, P. Moenne-Loccoz, M. T. Green, D. P. Goldberg, *J Am Chem Soc* **2017**, *139*, 13640-13643.

[20] D. Kass, T. Corona, K. Warm, B. Braun-Cula, U. Kuhlmann, E. Bill, S. Mebs, M. Swart, H. Dau, M. Haumann, P. Hildebrandt, K. Ray, *J Am Chem Soc* **2020**, *142*, 5924-5928.

[21] D. C. Lacy, R. Gupta, K. L. Stone, J. Greaves, J. W. Ziller, M. P. Hendrich, A. S. Borovik, *J Am Chem Soc* **2010**, *132*, 12188-12190.

[22] W. Rasheed, A. Draksharapu, S. Banerjee, V. G. Young, Jr., R. Fan, Y. Guo, M. Ozerov, J. Nehrkorn, J. Krzystek, J. Telser, L. Que, Jr., *Angew Chem Int Ed Engl* **2018**, *57*, 9387-9391.

[23] S. Schaub, A. Miska, J. Becker, S. Zahn, D. Mollenhauer, S. Sakshath, V. Schunemann, S. Schindler, *Angew Chem Int Ed Engl* **2018**, *57*, 5355-5358.

[24] A. Thibon, J. England, M. Martinho, V. G. Young, Jr., J. R. Frisch, R. Guillot, J. J. Girerd, E. Munck, L. Que, Jr., F. Banse, *Angew Chem Int Ed Engl* **2008**, *47*, 7064-7067.

[25] J. England, Y. Guo, E. R. Farquhar, V. G. Young, Jr., E. Munck, L. Que, Jr., *J Am Chem Soc* **2010**, *132*, 8635-8644.

[26] S. Meyer, I. Klawitter, S. Demeshko, E. Bill, F. Meyer, *Angew Chem Int Ed Engl* **2013**, *52*, 901-905.

[27] S. Sahu, B. Zhang, C. J. Pollock, M. Durr, C. G. Davies, A. M. Confer, I. Ivanovic-Burmazovic, M. A. Siegler, G. N. Jameson, C. Krebs, D. P. Goldberg, *J Am Chem Soc* **2016**, *138*, 12791-12802.

[28] J. England, J. O. Bigelow, K. M. Van Heuvelen, E. R. Farquhar, M. Martinho, K. K. Meier, J. R. Frisch, E. Munck, L. Que, Jr., *Chem Sci* **2014**, *5*, 1204-1215.

[29] W. Rasheed, R. Fan, C. S. Abelson, P. O. Peterson, W. M. Ching, Y. Guo, L. Que, Jr., *J Biol Inorg Chem* **2019**, *24*, 533-545.

[30] J. U. Rohde, J. H. In, M. H. Lim, W. W. Brennessel, M. R. Bukowski, A. Stubna, E. Munck, W. Nam, L. Que, Jr., *Science* **2003**, *299*, 1037-1039.

[31] E. J. Klinker, J. Kaizer, W. W. Brennessel, N. L. Woodrum, C. J. Cramer, L. Que, Jr., *Angew Chem Int Ed Engl* **2005**, *44*, 3690-3694.

[32] J. Prakash, G. T. Rohde, K. K. Meier, E. Munck, L. Que, Jr., *Inorg Chem* **2015**, *54*, 11055-11057.

[33] M. Lucic, D. A. Svistunenko, M. T. Wilson, A. K. Chaplin, B. Davy, A. Ebrahim, D. Axford, T. Tosha, H. Sugimoto, S. Owada, F. S. N. Dworkowski, I. Tews, R. L. Owen, M. A. Hough, J. A. R. Worrall, *Angew Chem Int Ed Engl* **2020**, *59*, 21656-21662.

[34] C. M. Casadei, A. Gumiero, C. L. Metcalfe, E. J. Murphy, J. Basran, M. G. Concilio, S. C. Teixeira, T. E. Schrader, A. J. Fielding, A. Ostermann, M. P. Blakeley, E. L. Raven, P. C. Moody, *Science* **2014**, *345*, 193-197.

[35] Y. T. Meharenna, T. Doukov, H. Li, S. M. Soltis, T. L. Poulos, *Biochemistry* **2010**, *49*, 2984-2986.

[36] G. Chreifi, E. L. Baxter, T. Doukov, A. E. Cohen, S. E. McPhillips, J. Song, Y. T. Meharenna, S. M. Soltis, T. L. Poulos, *Proc Natl Acad Sci U S A* **2016**, *113*, 1226-1231.

[37] H. P. Hersleth, T. Uchida, A. K. Rohr, T. Teschner, V. Schunemann, T. Kitagawa, A. X. Trautwein, C. H. Gorbitz, K. K. Andersson, *J Biol Chem* **2007**, *282*, 23372-23386.

[38] T. H. Yosca, R. K. Behan, C. M. Krest, E. L. Onderko, M. C. Langston, M. T. Green, *J Am Chem Soc* **2014**, *136*, 9124-9131.

[39] G. I. Berglund, G. H. Carlsson, A. T. Smith, H. Szoke, A. Henriksen, J. Hajdu, *Nature* **2002**, *417*, 463-468.

[40] K. L. Stone, R. K. Behan, M. T. Green, *Proc Natl Acad Sci U S A* **2005**, *102*, 16563-16565.

[41] M. T. Green, J. H. Dawson, H. B. Gray, *Science* **2004**, *304*, 1653-1656.

[42] M. Newcomb, J. A. Halgrimson, J. H. Horner, E. C. Wasinger, L. X. Chen, S. G. Sligar, *Proc Natl Acad Sci U S A* **2008**, *105*, 8179-8184.

[43] T. H. Yosca, M. C. Langston, C. M. Krest, E. L. Onderko, T. L. Grove, J. Livada, M. T. Green, *J Am Chem Soc* **2016**, *138*, 16016-16023.

[44] C. A. Bonagura, B. Bhaskar, H. Shimizu, H. Y. Li, M. Sundaramoorthy, D. E. McRee, D. B. Goodin, T. L. Poulos, *Biochemistry* **2003**, *42*, 5600-5608.

[45] G. N. Murshudov, A. I. Grebenko, J. A. Brannigan, A. A. Antson, V. V. Barynin, G. G. Dodson, Z. Dauter, K. S. Wilson, W. R. Melik-Adamyan, *Acta Crystallogr D Biol Crystallogr* **2002**, *58*, 1972-1982.

[46] H. P. Hersleth, B. Dalhus, C. H. Gorbitz, K. K. Andersson, *J. Inorg. Biochem.* **2002**, *7*, 299-304.
